# Carbon Emission Calculation and Influencing Factor Analysis Based on Industrial Big Data in the “Double Carbon” Era

**DOI:** 10.1155/2022/2815940

**Published:** 2022-02-28

**Authors:** Lu Zhang, Yan Yan, Wei Xu, Jun Sun, Yuanyuan Zhang

**Affiliations:** ^1^School of Management, Shenyang University of Technology, Shenyang, Liaoning Province, China; ^2^Journal Editorial Department, Shenyang University of Technology, Shenyang, Liaoning Province, China; ^3^Liaoning Urban and Rural Gas Co., Ltd, Liaoning Energy Investment (Group) Co., Ltd, Shenyang, Liaoning Province, China; ^4^Dalian Medical University, Dalian, Liaoning Province, China

## Abstract

The arrival of the “double carbon” era indicates that industrial carbon reduction with high energy consumption and high carbon emission is imperative. From the perspective of carbon emission driving factors, industrial carbon emission is decomposed into five influencing factors: energy intensity, energy structure, industrial structure, economic efficiency, and employee scale. Taking the data of 41 subindustries of industrial industry in Liaoning Province from 2010 to 2019 as the research sample, the carbon emission is calculated. The LMDI model is used to analyze and point out the difference in the driving contribution of carbon emissions of each subindustry. The results show that the total carbon emission of Liaoning province gradually decreases, decreases for the first time in 2014, and gradually turns from flat to upward. Economic efficiency is the only and most important reason to drive the increase of industrial carbon emissions in Liaoning Province, and energy efficiency is the primary factor to curb carbon emissions. High carbon industries play a significant role in promoting the increase of carbon emissions, while the medium and low carbon industries have a better effect on restraining carbon emissions. It provides reference and theoretical basis for the government to adjust the industrial structure, control industrial overcapacity, and realize the “double carbon” goal as soon as possible. It is of great significance for the country to optimize energy layout, ensure energy security, and implement the new energy strategy.

## 1. Introduction

As one of the most important material production departments in the national economy, industry is responsible for processing and assembling natural resources and raw materials, providing raw materials, fuel, and driving force for the industry itself and other sectors of the national economy, as well as providing industrial and industrial consumer goods for the people's material and cultural life. After the industrial revolution, with the large-scale development and use of fossil fuels such as coal, oil, and natural gas, the concentration of carbon dioxide in the Earth's atmosphere increased significantly, and the global carbon emission reached more than 34 billion tons in 2019. NASA observation data show that compared with the end of the 19th century, with the increase of global carbon emissions, the current global average temperature has also increased by more than 1.2oc, causing a series of extreme climate problems. More scientists predict that if this trend cannot be curbed, the glaciers in Antarctica will melt at the rate of 250–300 billion tons a year until they disappear completely, which will lead to a rise of at least 56 meters in the global sea level, which is a disaster for mankind. *∗∗∗* Countries must take decisive steps. China will increase its national independent contribution, adopt more effective policies and measures, strive to reach the peak of carbon dioxide emissions by 2030, and strive to achieve carbon neutrality by 2060. So far, the “double carbon” era has officially arrived, and carbon neutralization and carbon peak have also become the consensus of global green and sustainable development, marking the largest new industrial revolution since human society entered the industrial revolution. In the “double carbon” era, it is of great significance for the country to further adapt to the global trend and tide by measuring and decoupling the carbon intensity of various industries in order to better achieve the double carbon goal and point out the direction for the industry as the fundamental guarantee of national economic independence, political independence, and national defense modernization. According to statistics, the carbon emissions of the world's major carbon-emitting countries will reach 34.17 billion tons in 2019; among them, China's carbon emissions accounted for 28.76, the United States accounted for 14.53, and India accounted for 7.26, ranking among the top three; Russia, Japan, and Germany rank 4th, 5th, and 6^th^, respectively, with carbon emissions of 1.533 billion tons, 1.123 billion tons, and 684 million tons, respectively [1]. Among China's domestic provincial carbon emission intensity in 2019, Xinjiang, Shanxi, Inner Mongolia, and Ningxia have higher carbon emission intensity [2].

## 2. Literature Review

From the global perspective: Based on the data of 25 upper-middle-income countries from 1985 to 2014, Mujtaba et al. study the growth, correlation, and causality of kinked index among economic growth, energy consumption, population, trade openness, and carbon dioxide emissions. The results show that economic growth is negatively correlated with CO_2_ emissions and trade openness, respectively, while energy consumption is positively correlated with CO_2_ emissions and population. The determinants of carbon dioxide emissions are directly proportional to the country's CO2 emissions. There is a two-way causal relationship between population and economic growth, and trade openness and economic growth [3]. Georgia proposed that the Royal Court had revealed that in order to achieve net-zero carbon emissions, a series of measures would be taken, including banning some production materials and making 75% of food menus free of meat [4]. André and Valenciano-Salazar considered the environmental management system standards, the AHP method was used to analyze 24 companies and institutions in Costa Rica, and the results showed that the score of carbon neutrality (CN) is higher than the environmental management system standards. Therefore, they suggested that the adoption of environmental certification by organizations should be treated as a multistandard problem considering environmental sustainability, economy, and strategy [5].

From the industries perspective: Demetriou et al. considered that energy advantages and short driving distance make Cyprus a test field for an all-electric vehicle fleet, and they gave four different scenarios of minimum cost (LCSc), business as usual (BAU), carbon capture and storage (CCSc), and renewable energy (RESc) in parallel, deduced the hourly profile of weekly power production and consumption in winter and summer, and predicted that in 2050, RESc will have the highest power generation cost (0.115 euro/kWh) and the highest power loss rate (40%) and need 5600 mwh of domestic battery energy storage, and the current battery capacity in Europe is 3400 mwh [6]. Anonymous pointed out that the road map of the California Nevada Cement Association (CNCA) showed that by 2045, the three ways for the cement industry to realize net-zero carbon operation were to reduce production process emissions, reduce combustion emissions through fuel switching, and increase distributed power generation [7]. Srivastava et al. discussed the properties of available wastes and different strategies for decomposition or hydrolysis, and efficient microbial systems are emphasized. Some representative examples of biomass sources are used for biomass energy production by providing key information, as well as bioenergy production and environmental problems of plant organics. They also conducted efficient microbial and chemical process research on advanced biofuels extracted from organic matter, so as to promote the use of plant biomass to produce biofuels [8].

Comparing the literature above, the highlight of this paper is that we measure the carbon dioxide emissions of Liaoning industrial segments in different years and analyze the reasons for this phenomenon. At the same time, based on the LMDI decomposition method, we find out the real driving factors behind the growth of carbon dioxide emissions from industrial industries in Liaoning Province and give targeted suggestions from the macro and micro levels, so as to provide a method for the country to conduct horizontal comparison among provinces and vertical comparison among subdivided industries, which is of certain significance to achieve the goal of “double carbon” with high quality.

## 3. Research Method

### 3.1. Calculation Example of Carbon Strength

Carbon dioxide emissions can be divided into natural emissions and artificial emissions. Natural emission refers to the emission of carbon dioxide into the atmosphere from soil, ocean, forest, and other ecosystems; artificial emissions refer to carbon dioxide emissions caused by human activities, mainly from fossil energy consumption and biomass fuel combustion. There are three main research methods on carbon dioxide emissions by scholars at home and abroad, namely, emission coefficient method, material balance algorithm, and measurement method. Because the research object of this paper is industrial carbon emissions, it is mainly aimed at fossil energy, and the emission coefficient method is used to calculate. The calculation coefficients of various energy sources are shown in Table 1. The calculation formula is based on the reference formula proposed by the United Nations Intergovernmental Panel on climate change (IPCC) in 2006 (this part of the 2019 inventory guide has not changed) [10], combined with the availability and acceptability of variable data and the specific characteristics of national industries, and the calculation way is as follows:

(1) $$\begin{array}{c} {\text{CO}}_{2}=\sum_{i=1}^{N}{\left(\text{CO}2\right)}_{i}\\ =\sum_{i=1}^{N}E_{i}\times \mathrm{NC}V_{i}\times CC_{i}\times \mathrm{CO}F_{i}\times \frac{44}{12}, \end{array}$$

where *I* represents the type of energy; *E* is energy consumption; NCV is the average low calorific value; CC is carbon content; COF is carbon oxidation factor, expressed as a percentage of IPCC default value; 44 and 12 are the molecular weights of CO_2_ and C, respectively.

**Figure 1:**
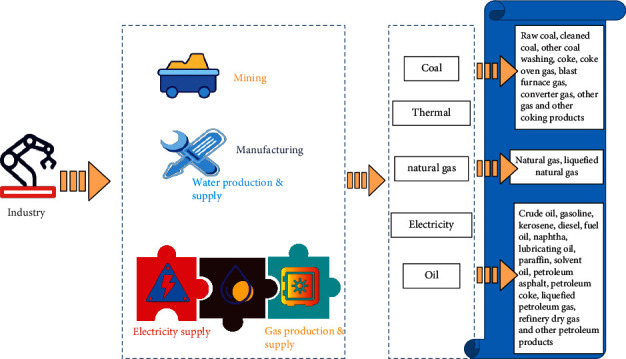
Industrial industry division and main energy consumption classification under national standards.

According to China's national standards, the division of industrial industries and the classification of main energy consumption are shown in Figure 1.

**Figure 2:**
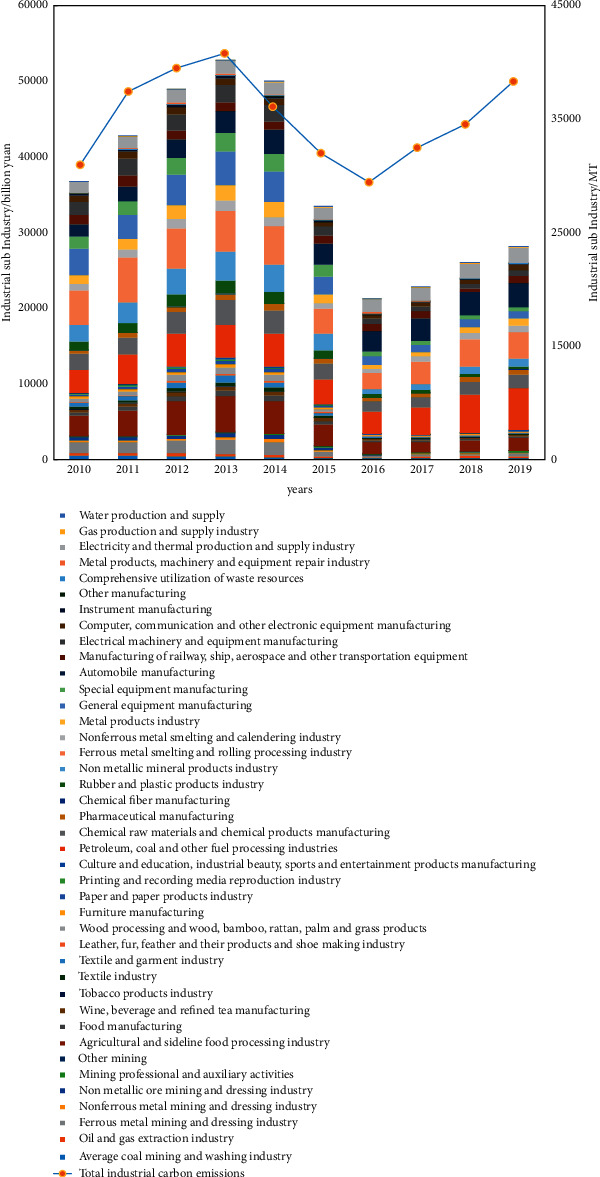
Change trend of total carbon emission and output value of various subindustries of Liaoning industry from 2010 to 2019.

Since *China's industrial Statistical Yearbook* will no longer publish a separate total industrial output value of subdivided industries after 2009, *Liaoning Provincial Statistical Yearbook* has an accurate calculation of the data. Considering the availability and accuracy of the data, 41 subindustries of the Liaoning industry from 2010 to 2019 are selected as the research object. The data on the energy consumption of kerosene and coke are short. In order to ensure the accuracy of the calculation, these two data are excluded, so five raw materials of fossil energy raw coal, gasoline, fuel oil, diesel, and natural gas are selected for calculation, and a few missing data are estimated and completed by linear interpolation method. Relevant data are from the 2006 IPCC guidelines for national greenhouse gas inventories, *Liaoning Statistical Yearbook* and *China Energy Statistical Yearbook*. After calculation and sorting, the change trend of total carbon emission and total industrial output value of each subindustry of Liaoning industry from 2010 to 2019 is shown in Figure 2, and the carbon emission is shown in Table 2 (considering comprehensive application, international practice is adopted).

### 3.2. Results' Analysis

In 2019, the industrial economy of Liaoning Province continued to optimize and upgrade the industrial structure along with the government's cultivation and growth of new industries and upgrading of traditional industries. However, affected by the escalation of Sino-US trade friction, the larger decline in the price of industrial products, the lack of domestic demand, and other factors, the industrial economy of Liaoning Province as a whole presents a situation of great difficulty in production and operation, slow pace, and falling profits. According to the calculation of carbon emissions of 41 industrial subindustries in Liaoning Province in recent ten years, the total carbon emissions of electric power, thermal power production, and supply industry and ferrous metal smelting and rolling processing industry are large, which are 162.0196 million T and 75.6808 million T, respectively, ranking first and second in 41 subindustries, accounting for 68.7% of the total carbon emissions of the whole industrial industry.

Compared with 2010, with the continuous progress of industrial production means, the downward trend of carbon emissions of most industries is more obvious. However, some industries still have the phenomenon of increasing carbon emissions instead of reducing carbon emissions. The largest increase is in the oil, coal, and other fuel processing industry, reaching 218.9%. The comprehensive utilization of waste resources followed, with an increase of 199.57%, and the carbon emission increased from 46700 t in 2010 to 139900 t in 2019. On the contrary, most equipment manufacturing industries have performed well, and the goal of energy conservation and emission reduction has been basically achieved through the joint action of various factors such as industrial upgrading, technological progress, and talent drive.

In 2019, the added value of industries above the designated size increased by 6.7% over 2018. Among them, the added value of the mining industry increased by 2.0% over the previous year, the added value of the manufacturing industry increased by 7.6%, and the added value of the high-tech manufacturing industry increased by 18.7%. The added value of power, heat, gas, and water production and supply also increased by 5.1% over last year.

In terms of specific industries, the added value of 25 of the 41 major industries maintained year-on-year growth, with a growth rate of 61.0%, and one industry was flat. Agricultural and sideline food processing industry increased by 7.1%, food manufacturing industry and textile industry decreased by 9.3% and 14.0%, respectively, the added value of petroleum, coal, and other fuel processing industry increased by 14.9%, the added value of chemical raw materials and chemical products manufacturing industry increased by 11.1%, pharmaceutical manufacturing industry increased by 9.1%, ferrous metal smelting and rolling processing industry, and nonferrous metal smelting and rolling processing industry increased by 5.0% and 3.3%, respectively, the nonmetallic mineral products industry increased by 2.7%, the general equipment manufacturing industry increased by 5.0%, and the special equipment manufacturing industry decreased by 2.1%; in terms of transportation, the automobile manufacturing industry increased by 2.5%, and the railway, ship, aerospace, and other transportation equipment manufacturing industry increased by 4.5%.

### 3.3. Analysis of Influencing Factors

Decomposing the core influencing factors of carbon emissions and studying the contribution of various factors to carbon emissions can not only provide theoretical reference for the government to formulate policies for phased economic and social green development but also provide a solid foundation for the country to achieve double carbon goals, maintain the bottom line of energy security, and lead high-quality development of the country. At present, the most commonly used methods for calculating carbon emissions are the following:

1. IPAT model: a quantitative model used to analyze the relationship between human activities and the environment. With the rapid growth of population and the progress of science and technology, people's pursuit of material life and the exploitation and utilization of resources have brought great pressure to the Earth's resources and environment. In other words, resources and environment are limited, but the human desire for material pursuit is unlimited. The model represents the pressure of human beings on the environment as the product of three factors, namely, the number of people, the degree of abundance, and the progress of science and technology, that is, *I* = *P* (Pop). × *A* (Aff) × *T* (Tech). Although the method is clear and simple in the calculation, it is not considered that environmental, resource, and social development are not linear in practice.
2. EKC: the environmental Kuznets curve, which was put forward by American economist Simon Smith Kuznets in 1955, mainly outlines the changes in the distribution of income along with the economic development process, also known as the inverted *U* curve. The formula is expressed as *E* = *β*_0_ + *β*_1_*Y* + *β*_2_*Y*^2^ + *µ.* Among them, *E* is the environmental pressure of the country or region, and *Y* is the economic output of the country or region. This method holds that when the economic development reaches a critical point, environmental pollution will tend to be stable no matter how the per capita income increases.
3. STIRPAT model: this method extends the IPAT model, adds randomness, and breaks through the limitation of unit single line assumption, and the calculation formula is expressed as *I*_*i*_ = *δ*_0_*P*_*i*_^*δ*1^*A*_*i*_^*δ*2^*T*_*i*_^*δ*3^*е*_*i*_.
4. LMDI model: compared with the first three methods, this method does not need to calculate with the help of an input-output table [11]. It is relatively simple to use, involves the nature of time series, and has more consistent aggregation, zero residual, and independent path [12]. Since the LMDI decomposition method has the advantages of complete decomposition, aggregation consistency, and path independence, this method is adopted in this paper.

The carbon emissions are decomposed in (1), as shown in the following:

(2) $$\begin{array}{c} C=\sum_{i=1}^{I}\sum_{j=1}^{J}C_{ij}\\ =\sum_{i=1}^{I}\sum_{j=1}^{J}\frac{C_{ij}}{E_{ij}}\times \frac{E_{ij}}{Y_{j}}\times \frac{E_{j}}{Y_{j}}\times \frac{Y_{j}}{Y}\times \frac{Y}{P}\times P, \end{array}$$

where *C*_*ij*_ represents the carbon emission of energy type *i* in *j* industry; *E*_*ij*_ represents the energy *i* consumption of *j* industry; *E*_*j*_ represents the total energy consumption of *j* industry; *Y*_*j*_ represents the total output value of the industrial *j* industry; *Y* represents the total industrial output value. ED_*ij*_ = *C*_*ij*_/*E*_*ij*_ represents the carbon emission intensity of energy *i* in industrial *j* industry; EM_*ij*_ = *E*_*ij*_/*E*_*j*_ represents the energy structure of energy *i* in industrial *j* industry; ET_*j*_ = *E*_*j*_/Y_*j*_ represents the energy efficiency of energy *i* in industrial *j* industry; ES_*j*_ = *Y*_*j*_/*Y* represents the industrial structure of the industrial *j* industry; EE = *Y*/*P* represents the economic efficiency of energy *i* in industrial *j* industry; *P* represents the number of employees. The decomposition formula of each carbon emission factor is calculated as shown in (3)–(8).

(3) $$\begin{array}{c} D_{\text{ED}}=\sum_{i=1}^{I}\sum_{j=1}^{J}\omega_{\mathrm{ij}}\;\ln\frac{{\text{ED}}_{ij}^{t+1}}{{\text{ED}}_{ij}^{t}}, \end{array}$$

(4) $$\begin{array}{c} D_{\text{EM}}=\sum_{i=1}^{I}\sum_{j=1}^{J}\omega_{\mathrm{ij}}\;\ln\frac{{\text{EM}}_{ij}^{t+1}}{{\text{EM}}_{ij}^{t}}, \end{array}$$

(5) $$\begin{array}{c} D_{\text{ET}}=\sum_{i=1}^{I}\sum_{j=1}^{J}\omega_{\mathrm{ij}}\;\ln\frac{{\text{ET}}_{j}^{t+1}}{{\text{ET}}_{j}^{t}}, \end{array}$$

(6) $$\begin{array}{c} D_{\text{ES}}=\sum_{i=1}^{I}\sum_{j=1}^{J}\omega_{\mathrm{ij}}\;\ln\frac{{\text{ES}}_{j}^{t+1}}{{\text{ES}}_{j}^{t}}, \end{array}$$

(7) $$\begin{array}{c} D_{\text{EE}}=\sum_{i=1}^{I}\sum_{j=1}^{J}\omega_{\mathrm{ij}}\;\ln\frac{{\text{EE}}_{i}^{t+1}}{{\text{EE}}_{i}^{t}}, \end{array}$$

(8) $$\begin{array}{c} D_{\text{EP}}=\sum_{i=1}^{I}\sum_{j=1}^{J}\omega_{\mathrm{ij}}\;\ln\frac{P_{j}^{t+1}}{P_{j}^{t}}. \end{array}$$

Among them, *ω*_*ij*_ is the weight of energy type *I* in J industry, and the calculation formula is shown in the following:

(9) $$\begin{array}{c} \omega_{ij}=L\left(C_{ij}^{t+1},C_{ij}^{t}\right)\\ =\frac{C_{ij}^{t+1}-C_{ij}^{t}}{\ln\;C_{ij}^{t+1}-\ln\;C_{ij}^{t}}. \end{array}$$

In view of using the two forms of “addition” and “multiplication” in the LMDI decomposition model, the final results are the same, and the addition is more intuitive and clear than “multiplication”, so “addition” is selected for factor decomposition in this paper, and the calculation formula is shown in the following:

(10) $$\begin{array}{c} {\text{IC}}^{t+1}－{\text{CI}}^{t}=D_{\text{ED}}+D_{\text{EM}}+D_{\text{ET}}+D_{\text{ES}}+D_{\text{EE}}+D_{\text{EP}}. \end{array}$$

### 3.4. Case Analysis

As a major industrial province, Liaoning has always undertaken the important task of providing technical equipment for various industries of China's national economy and national defense construction and has played an indispensable role in the whole process of industrialization. In 2020, faced with the impact of COVID-19 and the severe external environment of the world, Liaoning always adhered to the working keynote of steady progress. The industrial added value of above scale industries increased by 1.8% compared with that in 2019, of which the mining industry decreased by 0.6% compared with the same period last year, and the manufacturing industry grew by 1.8% over the same period last year. The electricity, water, gas, and aquatic industries and supply also increased by 3.6% over the same period last year.

Considering the national development of the green economy and the realization of the “double carbon” goal, Liaoning needs to balance economic growth and carbon emission, so take Liaoning as an example for LMDI decomposition analysis. In view of space, 41 industrial industries in Liaoning Province are numbered, as shown in Table 3. According to the carbon emissions of each subindustry, it is divided into high, medium, and low carbon industries, as shown in Table 4.

According to the LMDI model, 2010–2019 is selected as the research period to decompose the total industrial carbon emission in Liaoning Province and calculate the contribution degree and direction of five influencing factors such as energy intensity, energy structure, industrial structure, economic efficiency, and population effect to industrial carbon emission. The results are shown in Table 5.

In terms of energy intensity, from 2010 to 2019, the industrial energy intensity of Liaoning Province generally showed a downward trend, indicating that the industrial production technology of Liaoning Province has been improved to a certain extent. In terms of energy structure, from 2010 to 2019, the change of energy structure leads to the phenomenon of positive and negative alternation of industrial carbon emission year by year, with large fluctuation. The final cumulative carbon emission is −65.5186 million T, and compared with the other four influencing factors, the impact effect is the weakest, and the contribution rate is only 0.75%. In terms of industrial structure, most of the carbon emissions during the study period were negative, the cumulative carbon emissions decreased by 781259.98 million T, and the contribution rate to carbon emissions was 89.35%, indicating that it is the most important inhibitory factor of carbon emissions and plays a vital role in reducing carbon emissions in Liaoning Province. Economic efficiency is closely related to the total industrial output value, reflecting the progress of the economic level and technical means of the whole industry. As can be seen from Table 5, the cumulative increase of carbon emissions caused by economic efficiency has reached 1473.86 million T, which is the only driving factor leading to the increase of carbon emissions. The population effect reflects the attribute of social evaluation. During 2010–2019, the contribution rate to carbon emission reached 1.09%, second only to economic efficiency.

From 2010 to 2019, the cumulative carbon emission of industrial high carbon industries in Liaoning Province reached 39.15194 million T. The industry of comprehensive utilization of waste resources has the highest growth rate, accounting for 30.67% of the cumulative carbon emissions. The industry of oil, coal, and other fuel processing ranked second, with cumulative carbon emissions accounting for 29.65%. Among the influencing factors, most of the energy structure has a positive role in promoting, and only has a certain inhibitory effect on mining professionals and auxiliary activities; energy efficiency has the most obvious inhibitory effect on electricity, thermal production, and supply industry. The driving force of industrial structure on mining professional and auxiliary activities is relatively strong; economic efficiency and the size of employees have contributed a certain inhibitory force to the increase of carbon emissions in industrial high-carbon industries, but the emission reduction effect is also very weak in some industries, as shown in Table 6.

In the ten years from 2010 to 2019, the carbon emission of Liaoning's industrial carbon industry reached −1640377600 t. Carbon emissions from all industrial sectors have played a negative inhibitory role. From the perspective of influencing factors, the inhibition of energy structure is most significant, followed by energy efficiency indicators. The index of industrial structure has played a certain role in promoting the increment of industrial carbon emissions, as shown in Table 7.

During the study period, the cumulative carbon emissions of Liaoning's industrial low carbon industry reached −413557.2 million T. The driving force of promoting carbon emissions is strong in instrumentation manufacturing, gas production and supply, textile and clothing, and apparel industries. From the point of view of influencing factors, energy structure only has positive effects on gas production and supply industry and has played an inhibitory role in other low carbon industries. Energy efficiency has only played a catalytic role in the manufacturing of culture, education, industry, sports, and entertainment products, and the emission reduction effect of the remaining low carbon industries is outstanding. The three factors, including industrial structure, economic sub-Olympic Green, and the scale of employees, played a positive role in promoting the increase of carbon emissions in some low-carbon industries, but the impact was weak, as shown in Table 8.

## 4. Results and Discussion

From the perspective of the whole industrial sector, the total carbon emission of 41 industrial subindustries in Liaoning Province increased from 327372400 t to 360141300 t from 2010 to 2019, with an absolute increase of 1.1 times. From the perspective of evolution trend, the growth rate of total industrial carbon emission in Liaoning province gradually decreased from 2010 to 2013, and the carbon emission decreased for the first time after 2014. The decline rate of total carbon emission in Liaoning province gradually leveled off from 2014 to 2016, and there was an upward trend from 2016 to 2019.

From the analysis results of influencing factors and indicators, economic efficiency is the main and only reason driving the increase of carbon emissions from industrial industries in Liaoning Province; energy efficiency is the primary factor to restrain the carbon emission of industrial industry in Liaoning Province, followed by the industrial structure, while the impact of employee size and energy structure on the carbon emission of the whole industry is relatively weak.

From the perspective of the fine molecule industry, the high carbon industry plays an extremely significant role in promoting the increase of industrial carbon emissions, among which the driving role of comprehensive utilization of waste resources is the most prominent; medium and low carbon industries have good effects on restraining industrial carbon emissions in Liaoning Province. Therefore, the carbon emissions of the industrial sector mainly come from high carbon emission industries, so it is necessary to carry out energy conservation, emission reduction, upgrading, and transformation for subindustries.

To sum up, now that China has made clear the goal of “double carbon”, how to quickly unlock the carbon bondage has become the key to the next step. Future scholars can apply this method to the analysis of other regions or other industries to accurately eliminate the high dependence on fossil energy. Based on this, the government should refine the specific targets of carbon emissions by industry segments and build a green energy economic system, so as to reduce the consumption of fossil fuels. All industries should take the initiative to implement energy conservation and emission reduction and technological upgradings, such as carbon capture, storage, or negative emission technologies, so as to prevent carbon emissions from rising again and realize the two parallel rounds of optimizing the industrial structure and realizing the goal of “double carbon”.

## Figures and Tables

**Table 1:** Calculation correlation coefficient of various energy sources [9].

Energy name	Conversion coefficient of standard coal	Average low calorific value	Carbon content per unit calorific value (ton carbon/TJ)	Carbon oxidation rate	Carbon dioxide emission coefficient
Raw coal	0.7143 kgce/kg	20908 kJ/kg	26.37	0.94	1.9003 kg-CO_2_/kg
Gasoline	1.4714 kgce/kg	43070 kJ/kg	18.9	0.98	2.9251 kg-CO_2_/kg
Diesel oil	1.4571 kgce/kg	42652 kJ/kg	20.2	0.98	3.0959 kg-CO_2_/kg
Fuel oil	1.4286 kgce/kg	41816 kJ/kg	21.1	0.98	3.1705 kg-CO_2_/kg
Natural gas	1.3300 kgce/m^3^	38931 kJ/m^3^	15.3	0.99	2.1622 kg-CO_2_/m^3^

**Table 2:** Total carbon emissions of various subindustries of Liaoning industry from 2010 to 2019.

Industrial subindustry	2010 (MT)	2011 (MT)	2012 (MT)	2013 (MT)	2014 (MT)	2015 (MT)	2016 (MT)	2017 (MT)	2018 (MT)	2019 (MT)	Mean value (MT)
Average coal mining and washing industry	2819.37	3009.68	4618.05	2935.12	2617.01	2385.85	2312.94	2794.61	2837.36	2231.74	2856.17
Oil and gas extraction industry	445.39	567.75	452.97	481.03	303.20	299.39	278.40	312.68	328.80	286.56	375.62
Ferrous metal mining and dressing industry	130.28	188.98	156.51	156.45	127.46	121.11	54.91	71.83	98.30	101.52	120.74
Nonferrous metal mining and dressing industry	130.91	186.10	184.23	130.90	169.37	116.38	72.35	97.97	85.41	166.72	134.03
Nonmetallic ore mining and dressing industry	120.99	140.73	210.81	264.13	146.27	116.96	81.05	58.38	54.91	67.34	126.16
Mining professional and auxiliary activities	0.00	0.00	199.28	190.83	158.56	74.53	52.83	52.70	50.16	54.05	83.29
Other mining	0.56	0.56	0.00	0.00	0.00	0.00	0.00	0.00	0.00	0.00	0.11
Agricultural and sideline food processing industry	296.42	324.90	312.05	347.33	397.31	271.59	257.07	244.71	220.10	216.53	288.80
Food manufacturing	68.24	63.01	66.91	68.65	47.95	38.59	30.82	23.59	23.74	18.40	44.99
Wine, beverage, and refined tea manufacturing	96.84	94.40	95.12	84.06	70.58	55.75	57.84	41.28	35.46	29.47	66.08
Tobacco products industry	1.36	1.60	1.93	1.94	1.85	1.94	1.11	1.30	1.36	1.37	1.58
Textile industry	58.07	52.62	45.09	47.42	39.46	26.79	27.78	24.33	22.06	19.02	36.26
Textile and garment industry	66.31	64.22	54.49	50.43	42.62	20.67	21.87	12.47	5.97	5.47	34.45
Leather, Fur, feather, and their products and shoe-making industry	5.72	4.62	5.58	4.71	3.55	2.31	2.67	0.29	0.12	0.12	2.97
Wood processing and wood, bamboo, rattan, palm, and grass products	58.00	56.63	56.08	44.38	29.07	16.42	6.94	2.02	1.49	1.16	27.22
Furniture manufacturing	31.45	24.88	21.13	17.75	12.84	7.92	4.83	1.43	0.73	0.56	12.35
Paper and paper products industry	151.33	126.68	147.53	146.44	67.75	51.79	41.43	113.47	106.10	122.50	107.50
Printing and recording media reproduction industry	10.37	12.05	10.34	10.29	8.66	3.42	3.60	0.58	0.53	0.42	6.03
Culture and education, industrial beauty, sports, and entertainment products manufacturing	3.70	6.25	16.97	10.52	8.21	4.79	3.89	0.50	0.41	0.38	5.56
Petroleum, coal, and other fuel processing industries	1198.60	1396.47	1853.09	2101.50	2060.72	2110.48	2070.27	2353.59	2423.89	3822.34	2139.10
Chemical raw materials and chemical products manufacturing	1378.29	1487.34	1308.66	1342.52	1349.04	1048.84	835.01	758.02	487.04	516.22	1051.10
Pharmaceutical manufacturing	141.19	170.52	151.20	140.00	116.70	96.74	83.16	75.93	66.75	60.47	110.27
Chemical fiber manufacturing	97.33	89.01	86.30	81.48	67.78	77.62	74.84	63.52	10.27	9.87	65.80
Rubber and plastic products industry	213.13	232.42	215.56	212.53	141.94	118.78	61.42	31.59	27.80	27.11	128.23
Nonmetallic mineral products industry	2188.78	2270.55	3017.66	2876.19	2420.39	2015.23	1558.55	1622.11	1585.57	1992.49	2154.75
Ferrous metal smelting and rolling processing industry	7243.14	8029.31	7614.93	7745.26	7747.74	7501.76	7375.67	7235.43	7433.70	7753.88	7568.08
Nonferrous metal smelting and calendering industry	241.14	220.28	172.15	187.26	122.90	113.72	83.09	106.58	129.75	117.92	149.48
Metal products industry	119.49	136.20	145.51	172.42	78.89	50.72	42.61	28.36	73.75	82.56	93.05
General equipment manufacturing	318.81	363.12	316.55	360.04	207.16	192.36	44.86	17.03	14.70	12.93	184.76
Special equipment manufacturing	118.42	148.77	203.50	222.42	92.39	75.28	103.11	48.83	6.73	5.87	102.53
Automobile manufacturing	122.08	166.10	127.67	171.93	75.34	56.74	52.72	37.11	31.20	29.74	87.06
Manufacturing of railway, ship, aerospace, and other transportation equipment	104.98	84.32	127.74	210.81	28.94	19.82	22.13	21.70	53.33	11.91	68.57
Electrical machinery and equipment manufacturing	119.05	135.08	151.85	131.99	94.44	66.41	43.55	18.73	11.42	7.47	78.00
Computer, communication, and other electronic equipment manufacturing	13.62	13.19	10.69	16.13	7.22	5.10	4.67	2.95	2.74	2.07	7.84
Instrument manufacturing	13.40	14.92	14.81	19.91	4.27	2.64	3.27	1.34	1.03	0.98	7.66
Other manufacturing	26.86	41.05	18.94	10.88	2.38	1.61	2.09	0.64	0.12	0.14	10.47
Comprehensive utilization of waste resources	4.67	10.73	18.56	17.17	16.08	6.63	11.08	8.35	10.09	13.99	11.74
Metal products, machinery, and equipment repair industry	0.00	0.00	22.53	7.60	3.27	2.10	2.44	15.14	3.78	2.82	5.97
Electricity and thermal production and supply industry	14565.17	15651.38	14170.87	16059.87	16067.43	15954.85	16217.73	17062.14	18065.17	18204.99	16201.96
Gas production and supply industry	4.59	19.37	122.30	23.60	59.06	54.18	41.09	43.87	14.24	12.39	39.47
Water production and supply	9.19	13.97	13.98	14.51	7.37	8.44	8.75	5.52	3.79	2.64	8.82
Total industrial carbon emissions	32737.24	35619.76	36540.12	37118.4	35023.17	33196.25	32054.44	33412.62	34329.87	36014.13	

**Table 3:** Industrial code of Liaoning Province.

Number	Industry name
1	Average coal mining and washing industry
2	Oil and gas extraction industry
3	Ferrous metal mining and dressing industry
4	Nonferrous metal mining and dressing industry
5	Nonmetallic ore mining and dressing industry
6	Mining professional and auxiliary activities
7	Other mining
8	Agricultural and sideline food processing industry
9	Food manufacturing
10	Wine, beverage, and refined tea manufacturing
11	Tobacco products industry
12	Textile industry
13	Textile and garment industry
14	Leather, fur, feather, and their products and shoe-making industry
15	Wood processing and wood, bamboo, rattan, palm, and grass products
16	Furniture manufacturing
17	Paper and paper products industry
18	Printing and recording media reproduction industry
19	Culture and education, industrial beauty, sports, and entertainment products manufacturing
20	Petroleum, coal, and other fuel processing industries
21	Chemical raw materials and chemical products manufacturing
22	Pharmaceutical manufacturing
23	Chemical fiber manufacturing
24	Rubber and plastic products industry
25	Nonmetallic mineral products industry
26	Ferrous metal smelting and rolling processing industry
27	Nonferrous metal smelting and calendering industry
28	Metal products industry
29	General equipment manufacturing
30	Special equipment manufacturing
31	Automobile manufacturing
32	Manufacturing of railway, ship, aerospace, and other transportation equipment
33	Electrical machinery and equipment manufacturing
34	Computer, communication, and other electronic equipment manufacturing
35	Instrument manufacturing
36	Other manufacturing
37	Comprehensive utilization of waste resources
38	Metal products, machinery, and equipment repair industry
39	Electricity and thermal production and supply industry
40	Gas production and supply industry
41	Water production and supply

**Table 4:** Carbon emission classification of industrial subindustries in Liaoning.

Carbon emission classification	Subindustry name and number
High carbon	1, 4, 6, 7, 20, 37, 39
Medium carbon	2, 3, 5, 8, 9, 10, 11, 12, 17, 21, 22, 23, 24, 25, 26, 27, 28, 31, 32, 34
Low carbon	13, 14, 15, 16, 18, 19, 29, 30, 33, 35, 36, 38, 40, 41

**Table 5:** Effect decomposition results of influencing factors of industrial carbon emission in Liaoning Province from 2010 to 2019.

Year	*D*_EM_	*D*_ET_	*D*_ES_	*D*_EE_	*D*_EP_
2010-2011	−2182.06	−84889.77	−382022.83	1325.17	3842.70
2011-2012	2014.54	6223.20	15690.64	5010.07	−144.43
2012-2013	−1886.00	−6095.04	35906.64	823.19	1967.99
2013–2014	2124.50	1371.04	−24989.56	586.65	−2549.06
2014-2015	−795.55	2805.60	−54870.20	−10415.13	−3304.94
2015-2016	−2871.30	7341.45	−144101.15	−10683.17	−4059.59
2016–2017	2946.92	−1947.07	248635.79	5556.19	−3144.13
2017-2018	−3149.61	−3009.94	−425147.36	5739.87	−1425.34
2018-2019	−2753.30	−275.44	−50361.95	3531.02	−737.02
Cumulative value	−6551.86	−78475.97	−781259.98	1473.86	−9553.82

**Table 6:** Decomposition of influencing factors of high-carbon industry emission.

Industry number	*D*_EM_	*D*_ET_	*D*_ES_	*D*_EE_	*D*_EP_	Cumulative value
1	−1401.61	2278.35	−2808.45	1577.33	2742.41	2388.03
4	424.12	2576.38	−906.95	1120.25	−1592.19	1621.61
6	−27321.17	−7467.2	19408.79	−23655.51	43054.96	4019.87
7	45913.81	27694.5	−35603.48	−22145.29	−12710.51	3149.03
20	4390.79	853.83	4643.59	1670.32	48.94	11607.47
37	4653.54	16149.4	−10502.37	−5013.78	6719.32	12006.11
39	449.55	−13105.63	14880.7	2362.52	−227.32	4359.82

**Table 7:** Decomposition of influencing factors of medium-carbon industry emission.

Industry number	*D*_EM_	*D*_ET_	*D*_ES_	*D*_EE_	*D*_EP_	Cumulative value
2	−1954.24	−765.84	258.51	3187.93	−4809.65	−4083.29
3	−776.58	3674.68	−3017.86	−3529.69	−1017.05	−4666.5
5	−1892.08	3859.53	−4617.92	−2630.71	−2928.64	−8209.82
8	−1319.76	−5874.43	5950.63	−837.32	52.89	−2027.99
9	−5411.27	−3464.94	−634.47	−2346.2	−359.91	−12216.79
10	−4923.52	−8808.6	5251.45	−356.88	−2394.72	−11232.27
11	1292.57	−7236.2	−4804.68	−2046.37	−1234.21	−14028.89
12	−4618.68	3567.67	−6738.01	488.16	−6486.22	−13787.08
17	−1395.08	−1907.97	1760.38	−1232.53	−2123.94	−4899.14
21	−3990.99	−9009.1	6436.27	2718.01	−2713.72	−6559.53
22	−3678.45	−12873	10579.15	1154.72	274.17	−4543.41
23	−9047.62	493.57	−8218.17	3439.91	−5764.25	−19096.56
24	−8102.93	4797.24	−1944.47	−3106.02	−811.04	−9167.22
25	−667.85	2268.82	−1740.46	−794.77	−1195.08	−2129.34
26	−132.99	1179.06	−124.64	369.08	−1755.25	−464.74
27	−3354.22	−2214.37	318.9	936.92	−1143.38	−5456.15
28	−1344.72	−173.52	137.18	−498.4	378.39	−1501.07
31	−5946.8	−5804.74	1354.16	1029.88	2064.06	−7303.44
32	−9714.97	−7651.11	−1177.19	−353.38	−434.54	−19331.19
34	−7687.81	−12436.79	6090.77	1456.52	−756.03	−13333.34

**Table 8:** Decomposition of influencing factors of low-carbon industry emission.

Industry number	*D*_EM_	*D*_ET_	*D*_ES_	*D*_EE_	*D*_EP_	Cumulative value
13	−9693.74	−10924.28	2769.32	−1739.89	−2120.64	−21709.23
14	−15265.81	−6226.96	−7639.47	−4043.53	−1613.74	−34789.51
15	−15367.17	−9911.17	−4028.89	−4637.31	−2171.66	−36116.2
16	−16212.24	−10174.88	−4553.8	−3468.14	−1361.09	−35770.15
18	−12694.85	−11565.35	321.02	−2099.47	−2496.68	−28535.33
19	−8589.59	9440.41	−17278.95	−6669.27	−1132.25	−24229.65
29	−12602.5	−7342.06	−3784.34	−2731.48	−1214.46	−27674.84
30	−12099.44	−7934.57	−2922.32	−3774.22	−107.65	−26838.2
33	−11154.18	−10105.49	59.26	−2051.8	−1120.54	−24372.75
35	−10285.25	−8923.18	84.26	1173.68	−2219.48	−20169.97
36	−22319.71	−18089.79	−2479.32	−1848.59	−1658.37	−46395.78
38	−40122	−4609.37	623.25	−19603.4	34786.49	−28925.03
40	3602.46	−6215.82	−20250.59	835.61	768.47	−21259.87
41	−6163.57	−8547.37	−21957.44	213.62	−315.93	−36770.69

## Data Availability

The data used to support the findings of this study are available from the corresponding author upon request.
